# P-1616. Treatment Rates of Patients with Toxin Negative, PCR Positive *C.difficile* After a Change in Reflex Testing Order

**DOI:** 10.1093/ofid/ofae631.1783

**Published:** 2025-01-29

**Authors:** Marten Berke, Molly M Miller, Brook A Ringsdorf, Richard Hankins, Trevor C Van Schooneveld, Scott J Bergman

**Affiliations:** University of Nebraska Medical Center College of Pharmacy, Omaha, Nebraska; Nebraska Medicine, Bennington, Nebraska; Nebraska Medicine, Bennington, Nebraska; Nebraska Medicine, Bennington, Nebraska; University of Nebraska Medical Center, Omaha, NE; Nebraska Medicine, Bennington, Nebraska

## Abstract

**Background:**

On 12/11/23, our academic medical center changed from primarily GDH antigen/toxin A/B combination testing with reflex to *C. difficile* PCR in discordant samples (Figure 1) to PCR (either direct or from a multiplex gastrointestinal pathogen panel (GIPP)) as initial screening with positive specimens reflexed to antigen/toxin testing (Figure 2). After the algorithm change, we evaluated treatment rates for patients with positive PCR results who tested negative for *C. difficile* toxin.

**Figure:**
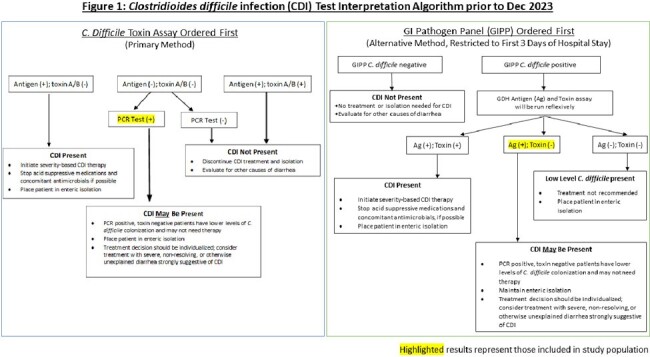

**Methods:**

New guidance for *C. difficile* infection (CDI) was posted on the Antimicrobial Stewardship website and education was provided to clinicians on the algorithm. Data was collected for inpatients ≥ 19 years old who were toxin negative, but antigen and PCR test positive for *C. difficile* from 10/1/22-3/31/24. GIPP could be ordered first during the entire period, but only in the first 3 hospital days. Treatment and recurrence rates for patients having a PCR test first were analyzed compared to rates for those with initial antigen/toxin tests. Treatment was counted if initiated within 2 days of test and continued ≥ 5 days, according to proposed NHSN definitions, and included oral vancomycin, fidaxomicin, or metronidazole if vancomycin was contraindicated. Based on estimated treatment rates, a sample size of 177 patients was targeted to detect a change from 80% to 60% treatment.

**Figure:**
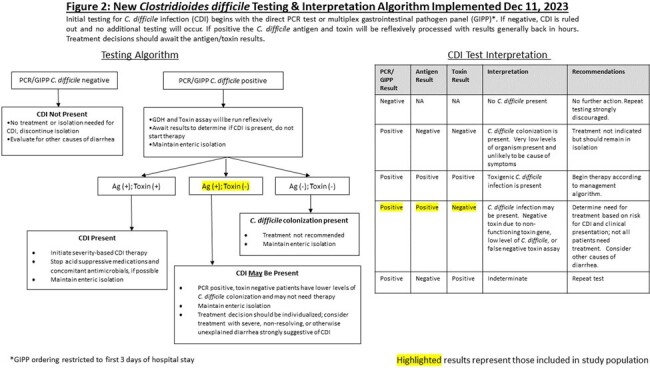

**Results:**

Out of 179 discordant CDI results prior to the testing change, treatment occurred in 151 (85.4%) cases. After the change, treatment was prescribed 26 out of 46 (56.5%) times, a 34% reduction (p< 0.01). Baseline characteristics were similar in each group, and 56.9% of tests were ordered in the first 3 days of hospital stay. Overall, when an antigen test was performed first and a positive PCR reported last, treatment occurred in 84 of 91 (92.3%) cases. When a PCR test was performed first and a negative toxin resulted last, treatment was prescribed in 93 of 134 (69.4%) cases (p< 0.01). Treatment was the same in each group (99% vancomycin). Recurrence rates were similar regardless of testing order, 5.4% with antigen first vs. 4.4% with PCR test first.

**Conclusion:**

Treatment rates for patients with discordant CDI results were significantly lower after the order of testing changed and when a negative toxin result was presented last.

**Disclosures:**

**Trevor C. Van Schooneveld, MD, FSHEA, FIDSA**, AN2 Therapeutics: Grant/Research Support|BioMerieux: Grant/Research Support|BioMerieux: Honoraria|Thermo-Fisher: Honoraria

